# Increased hospitalizations and economic burden in COPD with bronchiectasis: a nationwide representative study

**DOI:** 10.1038/s41598-022-07772-6

**Published:** 2022-03-09

**Authors:** Youlim Kim, Kyungjoo Kim, Chin Kook Rhee, Seung Won Ra

**Affiliations:** 1 Division of Pulmonary and Allergy, Department of Internal Medicine, Konkuk University Medical Center, Konkuk University School of Medicine, Seoul, Korea; 2 Division of Pulmonary, Allergy and Critical Care Medicine, Department of Internal Medicine, College of Medicine, Seoul St. Mary’s Hospital, The Catholic University of Korea, 222 Banpo-daero, Seocho-gu, Seoul, 06591 Republic of Korea; 3 Department of Internal Medicine, Ulsan University Hospital, University of Ulsan College of Medicine, 877 Bangeojinsunhwan-doro, Ulsan, 44033 Republic of Korea

**Keywords:** Diseases, Health care, Medical research

## Abstract

With the increasing use of computed tomography, bronchiectasis has become a common finding in patients with chronic obstructive pulmonary disease (COPD). However, the clinical aspects and medical utilization of COPD with bronchiectasis (BE) remain unclear. We aimed to investigate the BE effect on prognosis and medical utilization in patients with COPD. Among 263,747 COPD patients, we excluded patients lacking chest X-ray, CT, or pulmonary function test codes and classified 2583 GOLD-C/D patients matched according to age, sex, and medical aid as having COPD-BE (447 [17.3%]) and COPD without BE (2136 [82.7%]). Patients with COPD-BE showed a higher rate of acute exacerbation requiring antibiotics than those without BE. Moreover, multivariable analysis showed that BE co-existence was a crucial factor for moderate-to-severe exacerbation (incidence rate ratio [IRR] 1.071; 95% CI 1.012–1.134; *p* = 0.019). Patients with COPD-BE had a significantly higher rate of exacerbations requiring antibiotics, as well as treatment cost and duration (meant as number of days using hospitalization plus outpatient appointment), than those with COPD without BE (52.64 ± 65.29 vs. 40.19 ± 50.02 days, *p* < 0.001; 5984.08 ± 8316.96 vs. 4453.40 ± 7291.03 USD, *p* < 0.001). Compared with patients with COPD without BE, patients with COPD-BE experienced more exacerbations requiring antibiotics, more hospitalizations, and a higher medical cost.

## Introduction

Chronic obstructive pulmonary disease (COPD) is characterized by airflow limitation and is comprised of a complex disease group with varying effects and pathophysiologies. In patients with COPD, the bronchiectatic feature of airway wall thickening and dilatation is positively correlated with ageing and airflow obstruction severity^1,2^. With the increased use of computed tomography (CT), bronchiectasis (BE) has become a common finding among patients with COPD^3,4^. Moreover, a recent study using national patient sample data reported that 19.3% of Korean patients with BE presented with COPD as a comorbidity^5^.

It has been found that the prevalence of BE with COPD (COPD-BE) ranges from 25.6 to 69%^4,6–11^, with patients with COPD-BE presenting more exacerbations and worse lung function than those without BE. Further, a study on the Canadian Cohort of Obstructive Lung Disease (CanCOLD) reported that the BE prevalence in patients with mild-to-moderate and severe COPD was 14.1–22.2% and 35.1%, respectively^12^. In the CanCOLD cohort, COPD-BE was not associated with an increased risk of acute exacerbation; however, it was associated with dyspnoea and other respiratory symptoms. Other small-scale studies on COPD-BE have reported a wide prevalence range and varying characteristics with respect to exacerbation episodes and respiratory symptoms^4,12,13^. There is a need for a nationwide large-scale study to elucidate the clinical aspects, prognosis, and medical utilization in patients with COPD-BE.

Therefore, this nation-wide representative study aimed to investigate BE-associated outcomes and medical utilization in patients with COPD.

## Results

### Baseline characteristics of study population

**Figure 1:**
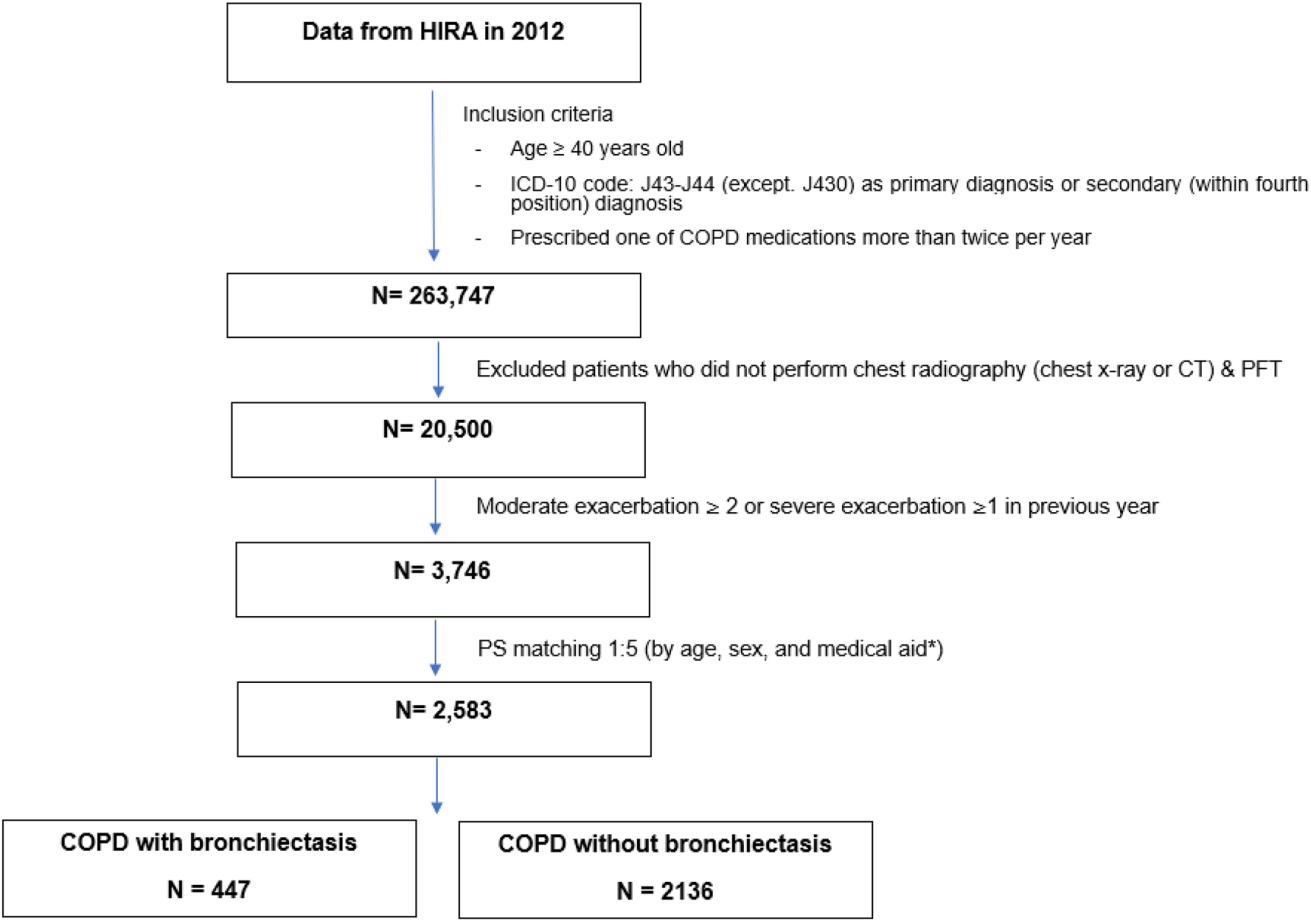
Flowchart of the study populations. *Patients with low socioeconomic status were covered with medical aid in Korea.

Among 263,747 patients with COPD, we excluded 243,247 participants who did not undergo chest X-ray/CT examination or a PFT and 16,754 participants without ≥ 2 exacerbations or ≥ 1 hospitalization within previous 1 year. Consequently, we enrolled the remaining 3746 GOLD C/D patients. Among them, 447 patients with COPD-BE underwent 1:5 PS matching according to age, sex, and medical aid while 2136 patients with COPD without BE were matched (Fig. 1). After PS matching, we analysed 2583 patients with GOLD C/D COPD. There was no significant between-group difference in age, sex, and medical aid. Table 1 presents the baseline characteristics of the two groups. The use of secondary (43.6% vs. 38.0%; *p* = 0.027) or tertiary hospitals (86.4% vs. 73.0%; *p* < 0.001) was higher in the COPD-BE group than in the COPD without BE group. Moreover, compared with the COPD without BE group, the COPD-BE group showed a higher number of moderate-to-severe exacerbations within the previous year (6.08 ± 6.73 vs. 5.42 ± 6.28; *p* = 0.047), prevalence of gastro-oesophageal reflux disease (43.6% vs. 38.2%; *p* = 0.031) and anaemia (10.3% vs. 7.3%; *p* = 0.032). Supplementary Table 1 showed the prescribed medications of each group.

**Table 1:** Baseline characteristics of study populations.

Variables	COPD with BE	COPD without BE	*p* value
Total	447	2136	
**Sex, male (%)**	328 (73.4)	1646 (77.1)	0.095
**Mean age (year)**	68.95 ± 9.91	69.62 ± 9.28	0.172
**Patients in each age group (%)**			
40–49 year	17 (3.8)	51 (2.4)	0.428
50–59 year	69 (15.4)	297 (13.9)	
60–69 year	122 (27.3)	600 (28.1)	
70–79 year	180 (40.3)	900 (42.1)	
> 80 year	59 (13.2)	288 (13.5)	
Medical aid*	326 (72.9)	1585 (74.2)	0.577
**Type of hospital use**			
Primary	213 (47.7)	1092 (51.1)	0.182
Secondary	195 (43.6)	812 (38.0)	0.027
Tertiary	386 (86.4)	1560 (73.0)	< 0.001
**Number of moderate to severe exacerbation in previous year**	6.08 ± 6.73	5.42 ± 6.28	0.047
**Chest X-rays**	435 (97.3)	2079 (97.3)	0.985
**Chest CTs**	58 (13.0)	217 (10.2)	0.079
**PFTs**	447 (100.0)	2136 (100.0)	–
**Co-morbidity**			
Ischemic heart disease	39 (8.7)	162 (7.6)	0.413
Congestive heart disease	81 (18.1)	349 (16.3)	0.358
Hypertension	238 (53.2)	1070 (50.1)	0.226
Diabetes mellitus	147 (32.9)	669 (31.3)	0.517
Hyperlipidemia	99 (22.2)	439 (20.6)	0.450
Osteoporosis	55 (12.3)	283 (13.3)	0.590
Depressive disorder	61 (13.7)	256 (12.0)	0.330
Gastro-esophageal reflux disorder	195 (43.6)	815 (38.2)	0.031
Pneumothorax	9 (2.0)	38 (1.8)	0.736
Arthritis	19 (4.3)	87 (4.1)	0.863
Anemia	46 (10.3)	156 (7.3)	0.032

Data are expressed as mean ± standard deviation or N (%).

Propensity score matching was performed in this study population, considering age, sex, and medical aid covering patients with low socioeconomic status.

*BE* bronchiectasis, *Chest CT* chest computed tomography, *PFT* pulmonary function test.

*Patients with low socioeconomic status were covered by medical aid in Korea.

### Exacerbation related clinical outcomes

Table 2 presents the between-group differences in the rate of moderate-to-severe exacerbation. Compared with the COPD without BE group, the COPD-BE group showed a significantly higher percentage of patients with moderate exacerbation who were prescribed with systemic steroids or antibiotics (84.8% vs. 77.7%, *p* < 0.001). Notably, compared with the COPD without BE group, the COPD-BE group showed a significantly higher percentage of patients with exacerbation who were only prescribed with antibiotics (51.7% vs. 38.0%, *p* < 0.001). Compared with the COPD without BE group, the COPD-BE group showed a higher percentage of patients with severe exacerbation requiring hospitalization and prescriptions of systemic steroids or antibiotics (76.5% vs. 63.0%, *p* < 0.001 for steroid/antibiotic prescriptions; 38.9% vs. 28.8%, *p* < 0.001 only antibiotic prescriptions). There were significant between-group differences in the percentage of patients with severe exacerbation requiring ICU care who were prescribed either systemic steroids or antibiotics (14.1% vs. 10.4%, *p* = 0.023) (Table 2). Multivariable analysis revealed that BE co-existence was a crucial factor for moderate-to-severe exacerbation (incidence rate ratio [IRR] 1.071, 95% CI 1.012–1.134, *p* = 0.019) (Table 3).

**Table 2:** Moderate to severe exacerbation related clinical outcomes.

Variables	COPD with BE (n = 447)	COPD without BE (n = 2136)	*p* value
**Patients with moderate exacerbation**			
AE that required systemic steroid or antibiotics	379 (84.8)	1659 (77.7)	< 0.001
AE that required systemic steroid only	229 (51.2)	132 (53.0)	0.497
AE that required antibiotics only	231 (51.7)	811 (38.0)	< 0.001
AE that required both	221 (49.4)	96 (42.9)	0.011
Patients with severe exacerbation*			
AE that required systemic steroid or antibiotics	342 (76.5)	1346 (63.0)	< 0.001
AE that required systemic steroid only	69 (15.4)	337 (15.8)	0.857
AE that required antibiotics only	174 (38.9)	614 (28.8)	< 0.001
AE that required both	276 (61.7)	1014 (47.5)	< 0.001
**Patients with severe exacerbation with ICU care**^**++**^			
AE that required systemic steroid or antibiotics	63 (14.1)	222 (10.4)	0.023
AE that required systemic steroid only	1 (0.2)	8 (0.4)	0.623
AE that required antibiotics only	19 (4.3)	64 (3.0)	0.172
AE that required both	46 (10.3)	170 (8.0)	0.105

Data are expressed as N (%).

*BE* bronchiectasis, *AE* acute exacerbation, *ER* emergency room, *ICU* intensive care unit.

*Severe exacerbation included the cases which admitted from emergency room.

^+^Moderate exacerbation was defined as outpatient department visit with a prescription of systemic steroids and/or antibiotics and severe exacerbation as emergency department visit or hospitalization with a prescription of systemic steroids and/or antibiotics.

^++^Severe exacerbation with ICU meant as worsening in symptoms and requiring ICU admissions.

**Table 3:** Factors affecting moderate to severe exacerbation.

Variables	Unadjusted			Adjusted		
	IRR	95% CI	*p* value	IRR	95% CI	*p* value
Age	0.992	(0.988, 0.996)	< 0.001	1.000	(0.998, 1.003)	0.842
Male	1.184	(1.082, 1.295)	< 0.001	1.023	(0.973, 1.083)	0.344
Medical aid*	0.909	(0.834, 0.990)	0.029	0.977	(0.929, 1.027)	0.362
Type of hospital use—tertiary	0.979	(0.896, 1.068)	0.629	1.047	(0.989, 1.108)	0.114
Number of moderate to severe exacerbation in previous year	1.071	(1.064, 1.077)	< 0.001	1.079	(1.075, 1.082)	< 0.001
Coexistence with BE	1.195	(1.082, 1.320)	< 0.001	1.071	(1.012, 1.134)	0.019
Any ICS use^+^	1.409	(1.286, 1.542)	< 0.001	1.093	(1.031, 1.158)	0.003
Any bronchodilators use^++^	1.158	(1.070, 1.254)	< 0.001	1.022	(0.972, 1.076)	0.393
Any steine use^‡^	1.591	(1.449, 1.747)	< 0.001	1.204	(1.134, 1.278)	< 0.001

*IRR* incidence rate ratio, *CI* confidence interval.

*Patients with low socioeconomic status were covered by medical aid in Korea.

^+^Any ICS use included ICS only and ICS plus LABA.

^++^Bronchodilator included LAMA, LABA.

^‡^Any steine included erdosteine, acetylcysteine, and carbocysteine.

### Medical utilization

Compared with the COPD without BE group, the COPD-BE group showed significantly more overall days of medical utilization (52.64 ± 65.29 vs. 40.19 ± 50.02 days, *p* < 0.001), which was mainly attributed to a high number of hospitalization days (39.14 ± 64.09 vs. 27.91 ± 49.70 days, *p* < 0.001) compared with the number of outpatient visits (13.50 ± 15.03 vs. 12.28 ± 13.54 days, *p* = 0.115). There was a similar between-group trend in the medical cost of hospitalization and outpatient visits (5586.30 ± 8232.74 vs. 4115.46 ± 7220.81 USD, *p* < 0.001; 397.78 ± 586.11 vs. 337.93 ± 697.80 USD, *p* = 0.058) (Table 4). Regression analysis revealed that gender (male), medical aid, visits to a tertiary hospital, and medications were significant factors (Table 5). Multivariable analysis of medical utilization revealed that co-existing BE was a significant factor in increasing medical use days (β = 7.304, *p* = 0.006) (Table 6).

**Table 4:** Medical cost of medical utilization.

Variables	COPD with BE	COPD without BE	*p* value
**Days of medical utilization (days)**			
Days of outpatient visits	13.50 ± 15.03	12.28 ± 13.54	0.115
Days of hospitalization	39.14 ± 64.09	27.91 ± 49.70	< 0.001
Total days	52.64 ± 65.29	40.19 ± 50.02	< 0.001
**Cost of medical utilization (USD)**			
Cost of outpatient visits	397.78 ± 586.11	337.93 ± 697.80	0.058
Cost of hospitalization	5586.30 ± 8232.74	4115.46 ± 7220.81	< 0.001
Total cost	5984.08 ± 8316.96	4453.40 ± 7291.03	< 0.001

Data are expressed as mean ± standard deviation.

*BE* bronchiectasis.

**Table 5:** Factors affecting medical costs (USD).

Variables	Unadjusted				Adjusted			
	β	SE	ST (β)	*p* value	β	SE	ST (β)	*p* value
Age	− 2.558	− 0.003	15.712	0.871	− 4.249	− 0.005	15.177	0.780
Male	1627.707	0.092	346.206	< 0.001	1052.628	0.059	337.514	0.002
Medical aid*	− 1871.488	− 0.110	334.375	< 0.001	− 1874.084	− 0.110	323.810	< 0.001
Type of hospital use—tertiary	4121.872	0.237	332.644	< 0.001	3088.448	0.178	335.534	< 0.001
Number of moderate to severe exacerbation in previous year	45.489	0.039	23.171	0.049	33.297	0.028	22.149	0.133
Coexistence with BE	1530.679	0.077	338.969	< 0.001	696.949	0.035	376.485	0.064
Any ICS use^+^	2424.591	0.135	349.149	< 0.001	1045.623	0.058	352.456	0.003
Any bronchodilators use^++^	2196.144	0.140	306.314	< 0.001	705.849	0.045	318.990	0.027
Any steine use^‡^	3318.497	0.180	356.515	< 0.001	2252.906	0.122	355.625	< 0.001

Data are expressed as mean ± standard deviation or N (%).

*Patients with low socioeconomic status were covered by medical aid in Korea.

^+^Any ICS use included ICS only and ICS plus LABA.

^++^Bronchodilator included LAMA, LABA.

^‡^Any steine included erdosteine, acetylcysteine, and carbocysteine.

**Table 6:** Factors affecting medical utilization (Days).

Variables	Unadjusted				Adjusted			
	β	SE	ST (β)	*p* value	β	SE	ST (β)	*p* value
Age	− 0.209	− 0.037	0.111	0.061	− 0.050	− 0.009	0.106	0.637
Male	11.994	0.096	2.453	< 0.001	8.807	0.070	2.348	< 0.0.01
Medical aid*	− 23.419	− 0.193	2.340	< 0.001	− 23.142	− 0.191	2.272	< 0.001
Type of hospital use—tertiary	16.894	0.137	2.405	< 0.001	8.930	0.072	2.494	< 0.001
Number of moderate to severe exacerbation in previous year	1.317	0.158	0.162	< 0.001	1.179	0.141	0.155	< 0.001
Coexistence with BE	12.443	0.089	2.755	< 0.001	7.304	0.052	2.641	0.006
Any ICS use^+^	17.650	0.139	2.474	< 0.001	10.270	0.08	2.473	< 0.001
Any bronchodilators use^++^	12.888	0.116	2.179	< 0.001	5.007	0.045	2.238	0.025
Any steine use^‡^	22.775	0.174	2.531	< 0.001	15.918	0.122	2.495	< 0.001

Data are expressed as mean ± standard deviation or N (%).

*Patients with low socioeconomic status were covered by medical aid in Korea.

^+^Any ICS use included ICS only and ICS plus LABA.

^++^Bronchodilator included LAMA, LABA.

^‡^Any steine included erdosteine, acetylcysteine, and carbocysteine.

## Discussion

This study investigated the effect of comorbid BE on clinical outcomes and medical utilization in patients with COPD. Our study yielded two main findings. First, compared with the COPD without BE group, the COPD-BE group showed has an increased risk of moderate-to-severe exacerbation and severe exacerbation requiring ICU admission. Second, the cost of medical utilization was significantly higher in patients with COPD with BE compared with patients without BE.

An emerging approach for COPD treatment is the determination of specific phenotypes. BE features in radiologic imaging is among the most prominent phenotypes and is frequently identified using the diagnostic assessment of chest CT scans. Previous studies have reported a COPD-BE prevalence widely ranging from 25.6 to 69%^4^; moreover, COPD-BE has revealed frequent exacerbations and poor prognoses^4,7,14^. However, most of these were small-scale studies with others not investigating the significance of COPD exacerbation with BE^7^. To our knowledge, this is the first study to analyse the relationship between COPD and BE using large-scale national claim data. The COPD-BE group showed an increased rate of moderate-to-severe exacerbation, especially severe exacerbation requiring hospitalizations and ICU admission. Moreover, BE coexistence was a significant risk factor for moderate-to-severe exacerbations.

Acute COPD exacerbation is generally caused by bacterial infections^15^. Ni et al.^4^ reported that the structural abnormalities of BE in patients with COPD can result in chronic colonization with potentially pathogenic micro-organisms, which causes recurrent infection, frequent exacerbations, and poorer prognosis. This can be prevented by the administration of effective antibiotics as additional treatment in patients with COPD-BE^16,17^. In our study, moderate and severe exacerbation events resulted in a significant increase in the rate of oral antibiotic prescription, which was consistent with previous findings^11^.

The present study investigated access to BE-related medical costs and clinical outcomes. A matched-cohort study on US health-care claim data analysed health care utilization in patients with COPD-BE^18^ and found that COPD-BE treatment involved greater medical resources (USD 15,685 [14,693–16,678]) than only treating COPD (USD 6262 [5655–6868]). These expenses were related to acute care management or the prescription of antibiotics, steroids, or bronchodilators at outpatient visits. In our study, we monitored medications, including ICS, bronchodilators, and oral medications, with the COPD-BE group showing a higher prescription than the COPD without BE group. Additionally, the percentage of prescribed systemic steroids or antibiotics was high in patients with hospitalizations, including ER visits and hospitalizations requiring ICU care. BE contributed to higher medical costs, particularly related to hospitalization. Additionally, factors associated with increased medical costs were tertiary hospital visits, as well as the prescription of ICS, bronchodilators, and mucolytic medications.

In our study, there was a significant occurrence of anaemia and GERD in the COPD-BE group. A previous study reported that the GERD prevalence was higher in the BE group, especially when accompanied with nontuberculous mycobacterial (NTM) disease^19^. Although the causal relationship remains unclear, one explainable hypothesis is that recurrent acid reflux irritates the airway, which causes NTM lung infection that leads to BE. Furthermore, in patients with COPD-BE, anaemia might result from systemic inflammation or erythropoiesis^20,21^. The anaemia prevalence is much higher in patients with COPD-BE due to the synergic effect of systemic inflammation.

This study has several limitations. First, COPD is diagnosed as having a postbronchodilator FEV_1_/FVC < 0.7 in the spirometry test^15^. However, this was a national cohort study using claim data and we could not determine the COPD diagnosis through lung function results. To make up for this limitation, the study population was defined using the operational definitions (ICD-10 code for COPD plus COPD drug prescription history), which were validated from previous studies using Korean National Health Insurance data^22–34^. Moreover, the inclusion of patients who underwent PFT and radiological examinations, including chest X-ray/CT, allowed more precise definition of the COPD and BE diagnoses. Previous studies have indicated that BE was more common among elderly patients with COPD with lower FEV_1_^4,35^. Since we could not determine each individual’s FEV_1_, we could not adjust the values. In case the COPD-BE group had lower FEV_1_ values than the COPD without BE group, we could not determine the causal effect of BE coexistence on increased exacerbation since the effect could have resulted from lower FEV1 values rather than BE coexistence in the COPD-BE group. Nonetheless, we attempted to adjust disease severity by including only GOLD C/D patients with COPD with exacerbations confirmed by ≥ 2 prescriptions of systemic steroids or antibiotics over one year. In the additional sensitivity analysis, even after including all patients with COPD, the COPD-BE group showed higher exacerbations and was an independent risk factor in multivariate analysis (data not shown). Second, potentially pathogenic micro-organisms can cause recurrent pulmonary infection^4,11^. Our study used Korean claim data; therefore, we could not determine the colonized pathogen in the COPD-BE group. Third, regression analysis revealed that any ICS or mucolytic use was associated with exacerbations. However, given that this was an observational study with cross-sectional analysis of 1-year insurance database rather than a cohort study with long-term follow-up, the ICS or mucolytics could have been prescribed more in patients with frequent exacerbations regardless of BE coexistence, which represents as a reverse causality issue. Traditionally, clinicians prescribe ICS and mucolytics for patients with a high exacerbation risk and bronchitis, respectively.

In conclusion, this study showed that patients with COPD-BE experienced a higher frequency of moderate-to-severe exacerbations than those with COPD only. Specifically, the COPD-BE group showed a higher rate of exacerbations requiring hospitalization, including ER visits and ICU care, which led to a high hospitalization rate with subsequently increased medical costs.

## Methods

### Study design and study population

We selected patients with COPD from the 2012 Health Insurance Review and Assessment (HIRA) database. COPD was identified as a major or minor diagnosis based on the International Classification of Disease Tenth Revision (ICD-10) code and defined according to the following criteria: (1) age ≥ 40 years; (2) ICD-10 codes for COPD (J43.x-J44.x, except J430) as the principal or secondary (within the fourth position) diagnosis; (3) use of > 1 medication for COPD at least twice per year [long-acting muscarinic antagonist (LAMA), long-acting beta-2 agonist (LABA), inhaled corticosteroids (ICS) plus LABA (ICS + LABA), LABA plus LAMA (LABA + LAMA), short-acting muscarinic antagonist (SAMA), short-acting beta-2 agonist (SABA), SABA plus SAMA (SABA + SAMA), phosphodiesterase-4 inhibitor (PDE4-I), systemic bronchodilator, or theophylline]. To increase the credibility of the recruited group, we identified GOLD C/D patients who underwent the pulmonary function test (PFT). Moreover, we performed propensity-score (PS) matching between the patients with COPD with and without BE according to age, sex, and medical aid covering patients with low socioeconomic status. BE was defined as at least one claim under the J47 ICD-10 code (except E84 [cystic fibrosis]) with the inclusion of only patients who underwent chest X-ray or chest CT examination to ensure BE diagnosis accuracy. The patients with COPD were allocated to the COPD- BE group and COPD without BE group. Additionally, comorbidities were defined using the following ICD-10 diagnosis codes: ischemic heart disease [angina pectoris (I20.x), myocardial infarction (I21.x, I22.x, or I25.2)], congestive heart disease (I50.x), hypertension (I10.x-I15.x), diabetes mellitus (E10.x-E14.x), hyperlipidaemia (E78.x), osteoporosis (M80.x-M81.x), depressive disorder (F32.x, F33.x), gastro-oesophageal reflux disorder [GERD] (K21.x), pneumothorax (J93.9), arthritis [osteoarthritis (M15.x-M19.x), rheumatoid arthritis (M05.x, M06.x)], and anaemia (D50.x). The frequency of moderate-to-severe exacerbation within the previous year was counted using 2011 HIRA data.

### Clinical outcomes and medical utilization measurement

In this study, the primary outcome was moderate-to-severe exacerbation. Moderate exacerbation was defined as an outpatient clinic visit with ICD-10 code for COPD (J43.x-J44.x, except J430) and prescription of systemic steroids and/or antibiotics. Severe exacerbation was defined as exacerbation requiring hospitalization or emergency room (ER) visits and prescription of systemic steroids and/or antibiotics. Additionally, severe exacerbation with a worsening condition resulting in admission to the Intensive care unit (ICU) was considered as severe exacerbation with ICU care.

We analyzed medical utilization and cost based on the 2012 HIRA database with the exclusion of medical utilization or costs not associated with COPD. We excluded patients with > 1 reimbursement per year for cancer (ICD-10 code: C00.x-C97.x), renal failure (N17.x-N19.x), and/or cerebrovascular disease (I60.x-I69.x) given the difficulty in distinguishing the significant expenses reimbursed for treatment of these diseases from those of COPD-related medical services. Regarding medication utilization and costs, we only analyzed COPD-related medications (LAMA, LABA, LABA + LAMA, ICS, ICS + LABA, SAMA, SABA, theophylline, LTRA, SABA + SAMA, PDE4-I, systemic steroids, systemic beta-agonist, mucolytics, and antitussive agent).

Regarding outpatient services, we only analyzed visits with a principal or secondary (within the fourth position) COPD diagnosis (J43.x-J44.x, except J430). For inpatient services, the analysis was limited to admission when the principal or secondary (within the fourth position) diagnosis was COPD (J43.x-J44.x, except J430) or a COPD-related disease (pneumonia: J12.x-J17.x; pulmonary thromboembolism: I26, I26.0, and I26.9; dyspnea: R06.0; or acute respiratory distress syndrome: J80). We collected data regarding age, sex, co-morbidity, hospitalizations, ER visits, ICU admissions, medical costs, and medication use. Further, we separately analysed the number of days that a particular outpatient and inpatient service was used (“used days”). All costs were presented in US dollars (USD) with an exchange rate of 1 USD for 1100 Korean won (exchange rate based on April 2018). This study was approved by the Institutional Review Board of Seoul National University Bundang Hospital (X-1801-447-907), which waived the requirement for informed consent since this study was based on anonymous health claim data. All methods were performed in accordance with the relevant guidelines and regulations.

### Statistical analysis

The selected patients were allocated to the COPD-BE group and COPD without BE group; moreover, we performed 1:5 PS matching according to age, sex, and socioeconomic status (insurance type). Between-group differences in categorical and continuous variables were assessed using the chi-square test and Student’s t-test, respectively.

To assess factors associated with moderate-to-severe exacerbations, we calculated the incidence rate using negative binomial regression and used the adjusting variables as age, sex, medical aid, type of hospital use (tertiary), number of moderate to severe exacerbation in previous year, coexistence with BE, any ICS use, any bronchodilators use, and any steine use. We performed multiple linear regression analysis to identify factors affecting medical costs by using the adjusting variables as age, sex, medical aid, type of hospital use (tertiary), number of moderate to severe exacerbation in previous year, coexistence with BE, any ICS use, any bronchodilators use, and any steine use. For linear regression analysis, we log-transformed the dependent variables since health care utilization variables are usually right-skewed (i.e., distributed with a long and heavy right tail). All tests were two-sided with statistical significance being set at *p* < 0.05. Data were expressed as mean ± standard deviation. All statistical analyses were performed using SAS version 9.2 software (SAS Institute Inc., Cary, NC, USA).

## Supplementary Information

Supplementary Information.
