# Supplementary material for: Increased hospitalizations and economic burden in COPD with bronchiectasis: a nationwide representative study
Source: Sci Rep. 2022 Mar 9;12:3829. doi: 10.1038/s41598-022-07772-6 (PMC8907167; doi:10.1038/s41598-022-07772-6)
Supplement: Supplementary file 1 — Supplementary Information. [file 41598_2022_7772_MOESM1_ESM.docx]

Supplementary table 1. Types and numbers of medication in COPD patients

| Medication | COPD with BE | COPD without BE | *p*-value |
| --- | --- | --- | --- |
| ICS | 211(47.2) | 793(37.1) | <0.001 |
| ICS+LABA | 327(73.2) | 1398(65.5) | 0.002 |
| LAMA | 329(73.6) | 1347(63.1) | <0.001 |
| LABA | 5(1.12) | 8(0.4) | 0.043 |
| LTRA | 232(51.9) | 1102(51.6) | 0.905 |
| OCS | 337(72.1) | 1539(72.1) | 0.150 |
| SAMA | 246(55.0) | 982(46.0) | <0.001 |
| SABA | 361(80.8) | 1604(75.1) | 0.011 |
| SABA+SAMA | - | 1(0.1) | - |
| Systemic beta agonist | 288(64.4) | 1299(60.8) | 0.153 |
| Theophylline | 410(91.7) | 1945(91.1) | 0.652 |
| Erdosteine | 259(57.9) | 1038(48.6) | <0.001 |
| Acetylcysteine | 310(69.4) | 1231(57.6) | <0.001 |
| Carbocysteine | 39(8.7) | 115(5.4) | 0.007 |
| Any steine use‡ | 397(88.8) | 1644(77.0) | <0.001 |

Note. Data are expressed as mean ± standard deviation or N (%), ICS, inhaled corticosteroids; ICS+LABA, ICS plus LABA; LAMA, long-acting muscarinic antagonist; LABA, long-acting beta-2 agonist; LTRA, leukotriene antagonist; OCS, oral corticosteroids; SAMA, short-acting muscarinic antagonist; SABA, short-acting beta-2 agonist; SABA+SAMA, SABA plus SAMA

‡Any steine included erdosteine, acetylcysteine, and carbocysteine.
